# Alterations in physique among young children after the Great East Japan Earthquake: Results from a nationwide survey

**DOI:** 10.1016/j.je.2016.09.012

**Published:** 2017-05-30

**Authors:** Masahiro Kikuya, Hiroko Matsubara, Mami Ishikuro, Yuki Sato, Taku Obara, Hirohito Metoki, Tsuyoshi Isojima, Susumu Yokoya, Noriko Kato, Toshiaki Tanaka, Shoichi Chida, Atsushi Ono, Mitsuaki Hosoya, Hiroshi Yokomichi, Zentaro Yamagata, Soichiro Tanaka, Shigeo Kure, Shinichi Kuriyama

**Affiliations:** a Tohoku Medical Megabank Organization, Tohoku University, Sendai, Japan; b Department of Molecular Epidemiology, Graduate School of Medicine, Tohoku University, Sendai, Japan; c Department of Disaster Public Health, International Research Institute of Disaster Science, Tohoku University, Sendai, Japan; d Department of Pediatrics, Graduate School of Medicine, The University of Tokyo, Tokyo, Japan; e Department of Medical Subspecialties, National Center for Child Health and Development, Tokyo, Japan; f Department of Early Childhood Care and Education, Jumonji University, Niiza, Japan; g Japanese Association for Human Auxology, Tokyo, Japan; h Department of Pediatrics, School of Medicine, Iwate Medical University, Morioka, Japan; i Department of Pediatrics, School of Medicine, Fukushima Medical University, Fukushima, Japan; j Department of Health Sciences, School of Medicine, University of Yamanashi, Chuo, Japan; k Department of Pediatrics, Graduate School of Medicine, Tohoku University, Sendai, Japan

**Keywords:** Earthquake, Tsunami, Physical growth, Childhood obesity

## Abstract

**Background:**

Data for earthquake-related alterations in physique among young children in developed countries is lacking. The Great East Japan Earthquake caused severe damage in Iwate, Miyagi, and Fukushima Prefectures in northeastern Japan.

**Methods:**

We retrospectively obtained anthropometric measurements in nursery school from 40,046 (cohort 1, historical control) and 53,492 (cohort 2) children aged 3.5–4.5 years without overweight in October 2008, and in October 2010, respectively. At the time of the earthquake in March, 2011, children in cohort 1 had already graduated from nursery school; however, children in cohort 2 were still enrolled in nursery school at this time. We compared the onset of overweight at 1 year after the baseline between children enrolled in their school located in one of the three target prefectures versus those in other prefectures using a logistic regression model, with adjustment for sex, age, history of disease, and obesity index at baseline. Overweight was defined as an obesity index of >+15%, which was calculated as (weight minus sex- and height-specific standard weight)/sex- and height-specific standard weight.

**Results:**

The odds ratio (OR) for the onset of overweight in the three target prefectures was significant in cohort 2 (OR 1.25; 95% confidence interval [CI], 1.01–1.55) but not in cohort 1. When the two cohort were pooled (n = 93,538), the OR of the interaction term for school location × cohort was significant (OR 1.56; 95% CI, 1.09–2.23).

**Conclusions:**

Incident overweight in young children was significantly more common in the three prefectures affected by the Great East Japan Earthquake than in other prefectures after the disaster.

## Highlights

• We obtained anthropometric data from 93,538 children aged 3.5–4.5 years.
• Anthropometric data were measured before and after the Great East Japan Earthquake.
• We compared the data in Iwate, Miyagi, and Fukushima vs. the other prefectures.
• Incident overweight was higher in the three prefectures than the other prefectures.

## Introduction

**Fig. 1:**
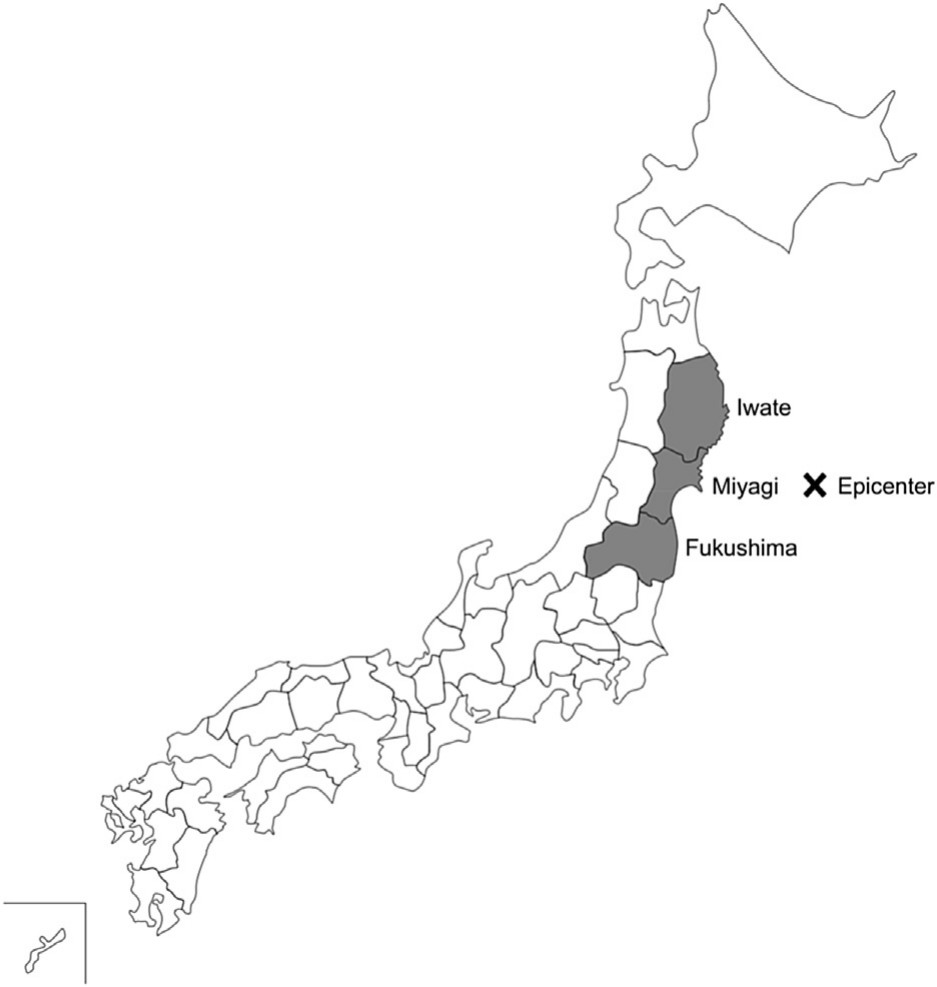
Map of Japan showing the epicenter of the Great East Japan Earthquake. Iwate, Miyagi, and Fukushima Prefectures were the areas most severely damaged by the earthquake.

Humanity has suffered many indiscriminate disasters throughout history. Disasters can occur anywhere in the world, not only in developing countries but also in developed countries, where advanced medical support systems might contribute to the acute recovery of survivors' health. We would like to share our experience from the Great East Japan Earthquake of March 11, 2011 with people around the world to help reduce disaster risk, especially the prolonged impact of this huge earthquake on children's health in Japan, one of the most developed countries in the world. The Great East Japan Earthquake was followed by a giant tsunami that caused severe damage along the Pacific coast of Northeastern Japan, with deaths or missing persons amounting to 18,466 in the three prefectures most affected by the earthquake: Iwate, Miyagi, and Fukushima (Fig. 1).^1^

Disasters affect children's health by increasing the prevalence of psychiatric problems^2^, ^3^ and physical symptoms.^4^, ^5^, ^6^, ^7^ In particular, alterations in physique have been a major health problem. In developing countries, underweight among children after disasters has been recognized as a serious health problem.^6^, ^7^ However, in developed countries, there are no data on earthquake-related alterations in physique among young children. Extrapolation of results from developing countries to developed countries may not be appropriate, since the social environment, including financial resources, educational background of parents, development of transportation networks, and public health policies and resources, are distinctly different between developing and developed countries.

We conducted a nationwide survey to investigate the possible health impacts of the Great East Japan Earthquake on nursery school children.^8^ According to childcare guidelines, all nursery schools in Japan must perform periodic body measurements.^9^ Our survey retrospectively collected pre-existing data for scheduled anthropometric measurements accumulated in each school before and after the earthquake. The aim of the present analysis was to clarify alterations in physique among young children after a huge earthquake in a developed country.

## Methods

### Design

The present study is part of the “Surveillance Study on Child Health in the Great East Japan Earthquake Disaster Area”.^8^ In this surveillance study, children's physical development data (i.e., height and weight) for a maximum of 7 years were retrospectively collected for the purpose of investigating the impact of the Great East Japan Earthquake on the physical development and physical and mental health of children.^8^ From all 47 prefectures in Japan, 23,711 nursery schools certified by the governor of each prefecture were asked to join the study. Among them, 4266 schools showed interest in taking part, and study questionnaires were mailed to these schools. Of these, 3624 schools returned completed questionnaires. The participation rate was 15.3% in total and was significantly different between the three prefectures most impacted by the earthquake (30.3%) and the other prefectures (14.6%, chi-square *P* < 0.0001). The participation rates were 22.6% in Iwate, 38.2% in Miyagi, and 30.6% in Fukushima.^8^ The survey was conducted from September 2012 through December 2012.

The survey protocol was approved by the institutional review board of Tohoku University. It was not necessary to obtain informed consent from the children and their parents because any information that could possibly identify individuals, such as names and addresses, was not collected. Furthermore, the purposes and procedures of the survey were explained to nursery teachers in the invitation letter and announced to parents via a poster displayed in each nursery school. Parents had the right to opt out of the study. The information for the present study was also disclosed to the public on the website for the Graduate School of Medicine, Tohoku University, Sendai, Japan.

### Study population

From the 3624 nursery schools that participated in the “Surveillance Study on Child Health in the Great East Japan Earthquake Disaster Area”,^8^ data for two groups of young children born in different fiscal years were collected for the present study. We obtained data from 54,558 children who were born from April 2, 2004 to April 1, 2005 (cohort 1) and from 69,702 children who were born from April 2, 2006 to April 1, 2007 (cohort 2).^8^ The reason for selecting these periods was that new school terms in Japan start on April 1 and the birth dates for the children in each class range from April 2 of the current year to April 1 of the following year. Participants in cohort 1 were children aged 5 years in 2010 who did not experience the earthquake during their nursery school days. Consequently, cohort 1 was a historical control group. On the other hand, participants in cohort 2 were children aged 5 years in 2012 who did experience the earthquake during their nursery school days. According to previous cross-sectional reports, children in northeastern Japan, including Iwate, Miyagi, and Fukushima Prefectures, show a higher prevalence of obesity than other parts of Japan.^10^ To separate the regional differences and the effects of the earthquake on children, as well as to collect historical control data (cohort 1), we decided to collect data from all over Japan, not only from the earthquake-affected prefectures, using a large sample.

**Fig. 2:**
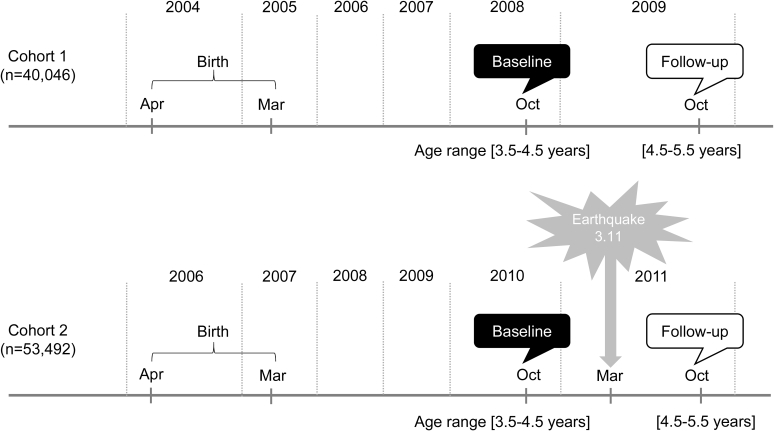
Study timeline. Study subjects in cohort 1 were young children born from April 2, 2004 to April 1, 2005, and were attending nursery school classes for 5-year-olds in 2010; cohort 1 did not experience the earthquake during their nursery schools days. In contrast, study subjects in cohort 2 were born from April 2, 2006 to April 1, 2007, and were attending nursery school classes for 5-year-olds in 2012; cohort 2 experienced the earthquake during their nursery schools days. Thus, cohort 1 was a historical control of the earthquake. The baseline survey for both cohorts was performed when the children were aged 3.5–4.5 years (i.e., in October 2008 for cohort 1 and in October 2010 for cohort 2). The follow-up survey was performed 1 year after baseline (i.e., in October 2009 for cohort 1, and October 2011 for cohort 2).

As initial data cleaning, we excluded data for children who were not born in the target fiscal year and children whose anthropometric measurements were not provided, leaving a total of 53,747 children in cohort 1 and 69,004 children in cohort 2.^8^ We then excluded 1187 and 1362 children from cohort 1 and cohort 2, respectively, due to the following reasons: (1) sex was unknown (n = 475 and 584, respectively), (2) birth year or month was unknown (n = 392 and 423, respectively), (3) measurement data were null in individual children after deleting overlapping measurements or inconsistent height measurements that were smaller than the previous value (n = 125 and 75, respectively), (4) duplicated data in different children (n = 1 and 0, respectively), and (5) children who had more than one height measurement that exceeded +3 standard deviations (SD) (n = 194 and 280, respectively). Therefore, the number of children in cohort 1 and cohort 2 were 52,560 and 67,642, respectively. For analysis of overweight, we further excluded 12,514 and 14,150 children from cohort 1 and cohort 2, respectively, because of missing height or weight at baseline (n = 9811 and 11,158, respectively) and at follow-up (n = 1024 and 993, respectively), and those who were overweight at baseline (n = 1679 and 1999, respectively). Therefore, a total of 40,046 children in cohort 1 and 53,492 children in cohort 2 were included in the final analysis for overweight. For analysis of underweight, we excluded children who were underweight at baseline (n = 409 in cohort 1 and 580 in cohort 2), instead of excluding those with overweight at baseline. The definition of baseline, follow-up, overweight, and underweight is explained below in the data analysis section (Fig. 2).

### Data collection

We used different questionnaires for cohort 1 (questionnaire B1) and cohort 2 (questionnaire B2).^8^ Both questionnaires were completed by nursery school teachers, commonly contained individual children's data, and were anonymous questionnaires that included items on sex, birth year, birth month, height in centimeters, and weight in kilograms, as well as history of disease diagnosed by a medical doctor. In both questionnaires, data for height and weight were collected in April and October, and then subsequently every 6 months from April 2004 to October 2010 for questionnaire B1 and from April 2006 to October 2012 for questionnaire B2. According to Japanese guidelines for childcare,^9^ all nursery schools perform periodic anthropometric measurements, usually once a month, following the measurement procedure recommended by the Ministry of Health, Labour and Welfare.^11^ We retrospectively collected individual children's height and weight as measured in April and October. Questionnaire B2, which was for children in cohort 2 who ranged in age from 3.9 to 4.9 years at the time of the earthquake, also collected data on the type of severe disaster experience (“house destroyed”, “tsunami”, “fire”, “moving house”, “living in an evacuation center”, “death in the family”, and “others”). However, we did not ask about the experience of the earthquake itself in the questionnaire, because almost all inhabitant living in the three prefectures obviously experienced the earthquake.^12^

### Data analysis

We defined overweight and underweight as an obesity index of >+15% and <−15%, respectively, according to the children's physical development assessment manual,^11^ which is available on the official website of the Ministry of Health, Labour and Welfare, Japan. The obesity index (%) was calculated as (weight in kilograms – standard weight in kilograms)/standard weight in kilograms × 100. The standard weight was defined as 0.00206 × (height in centimeters)^2^–0.1166 × (height in centimeters) + 6.5273 for boys, and 0.00249 × (height in centimeters)^2^–0.1858 × (height in centimeters) + 9.0360 for girls.^11^

In cohort 2, we set baseline and follow-up surveys as October 2010 and October 2011, which corresponded to before and after the Great East Japan Earthquake, respectively (Fig. 2). Then, we compared the onset of overweight or underweight at follow-up between the three prefectures of Iwate, Miyagi, and Fukushima compared with other prefectures in Japan among children without overweight or underweight at baseline, respectively. The children ranged in age from 3.5–4.5 years old at baseline and 4.5–5.5 years old at follow-up. We performed multivariate logistic regression analysis for the onset of overweight (present = 1, absent = 0) as the dependent variable, and school location (the three affected prefectures = 1, other prefectures = 0), sex (boy = 1, girl = 0), age (continuous variable), history of disease (present = 1, absent = 0), and obesity index at baseline (continuous variable, per 1% increase) as independent variables.

For cohort 1, the historical control, we performed similar analyses using data for the same months 2 years prior to the data for cohort 2 (i.e., the baseline was October 2008, and the follow-up was October 2009), which also corresponded to the ages of 3.5–4.5 years old at baseline and 4.5–5.5 years old at follow-up (Fig. 2).

Then, we pooled data from the two cohorts to more precisely estimate the effect of the earthquake on odds of overweight using a single logistic regression model that included an interaction term for school location (the three affected prefectures = 1, the other prefectures = 0) and cohort (Cohort 2 = 1, Cohort 1 = 0). We further performed a permutation test to investigate whether the effect of the earthquake was unique to the three affected prefectures. We assigned a random three prefectures as an exposure group, and calculated odds ratio for interaction term for exposure group × cohort. We repeated this analysis 1000 times to generate 1000 estimates of the odds ratios of the interaction term of exposure group × cohort. To the distribution of odds ratios of the interaction term, we compared the odds ratio of the interaction term for the three affected prefectures (Iwate, Miyagi, and Fukushima Prefectures) × cohort.

We compared means and proportions using the unpaired *t*-test or analysis of variance, as well as the chi-square test. *P* values less than 0.05 for a two-sided test were considered to be statistically significant. All statistical analyses were performed using SAS (version 9.4; SAS Institute Inc., Cary, NC, USA).

## Results

Table 1 shows the characteristics of the children at baseline. In both cohort 1 and cohort 2, the prevalence of boys and age were similar between the three prefectures of Iwate, Miyagi, and Fukushima compared with other prefectures in Japan. However, history of disease was significantly higher in the three affected prefectures than in the other prefectures (14.4% vs. 10.9% in cohort 1 and 15.3% vs. 11.0% in cohort 2, both *P* < 0.0001). Obesity index and body mass index (BMI) were significantly higher in the three affected prefectures than the other prefectures. Data for severe disaster experience were only collected in cohort 2, with 49,174 responses collected. Severe disaster experiences were reported by 369 children (11.6%) in the three affected prefectures. On the other hand, only 0.29% of the children in other prefectures had severe disaster experience.

**Table 1:** Characteristics of young children at baseline by school location (in Iwate, Miyagi, and Fukushima Prefectures vs. other prefectures in Japan).

	Cohort 1 (n = 40,046)		Cohort 2 (n = 53,492)	
	Three prefectures (n = 2555)	Other prefectures (n = 37,491)	Three prefectures (n = 3551)	Other prefectures (n = 49,941)
Boys	1383 (54.1%)	19,582 (52.3%)	1843 (51.9%)	25,976 (52.0%)
Age, years	4.06 (0.28)	4.05 (0.29)	4.06 (0.29)	4.05 (0.29)
History of diseases	367 (14.4%)*	4086 (10.9%)	544 (15.3%)*	5474 (11.0%)
Atopic dermatitis	95 (3.7%)	1403 (3.7%)	133 (3.8%)	1662 (3.3%)
Asthma	89 (3.5%)^†^	1035 (2.8%)	167 (4.7%)*	1526 (3.1%)
Heart disease	10 (0.4%)	136 (0.4%)	22 (0.6%)^†^	177 (0.4%)
Kidney disease	2 (0.1%)	12 (0.0%)	2 (0.1%)	29 (0.1%)
Other disease	229 (9.0%)*	1990 (5.3%)	277 (7.8%)*	2707 (5.4%)
Obesity index, %	0.66 (6.55)*	−0.33 (6.50)	0.29 (6.53)*	−0.59 (6.52)
BMI, kg/m^2^	15.5 (1.0)*	15.4 (1.0)	15.5 (1.0)*	15.4 (1.0)
Severe disaster experience^‡^	N/A	N/A	369 (11.6%)*	134 (0.29%)
House destroyed^‡^	N/A	N/A	187 (5.9%)*	22 (0.05%)
Tsunami^‡^	N/A	N/A	97 (3.0%)*	12 (0.03%)
Fire^‡^	N/A	N/A	1 (0.03%)	0 (0.0%)
Moving house^‡^	N/A	N/A	50 (1.6%)*	4 (0.01%)
Evacuation center^‡^	N/A	N/A	89 (2.8%)*	16 (0.03%)
Death in the family^‡^	N/A	N/A	17 (0.5%)*	0 (0.0%)
Others^‡^	N/A	N/A	93 (2.9%)*	87 (0.2%)

Categorical variables are indicated as numbers (percent). Continuous variables are indicated as mean (standard deviation).

*P < 0.0001.

^‡^*P* < 0.05 between Iwate, Miyagi, and Fukushima Prefectures vs. the other prefectures using Fisher's exact test, chi-square test, or unpaired *t*-test. ^‡^Data for severe disaster experience were collected only for cohort 2, with 49,174 responses collected.

We performed a univariate analysis of the onset of overweight at follow-up among children without overweight at baseline. In cohort 1, the onset of overweight was similar between the three affected prefectures and other prefectures (number of events/population = 60/2555, 2.35% vs. 815/37,491, 2.17%; *P* = 0.53). However, in cohort 2, the onset of overweight was significantly higher in the three affected prefectures than in the other prefectures (115/3551, 3.24% vs. 1120/49,941, 2.24%, *P* = 0.0003). The significant difference in the odds ratio of overweight between the three affected prefectures and the other prefectures remained even after multiple adjustments for possible confounding factors (Table 2). The odds ratio of overweight in the three affected prefectures was significant only in cohort 2 (odds ratio 1.25; 95% confidence interval [CI], 1.01–1.55), but not in cohort 1 (odds ratio 0.80; 95% CI, 0.60–1.06), after adjustments were applied for sex, age, history of disease, and obesity index at baseline (Table 2). In the pooled data from the two cohorts, the odds ratio of the interaction term of school location × cohort was significantly high (odds ratio 1.56; 95% CI, 1.09–2.23), but the odds ratio in the three affected prefectures was no longer significant (Table 2). Then, we performed a permutation test for the odds ratio of the interaction term for three random prefectures × cohort and obtained a normal distribution of the odds ratio (*P* value of Kolmogorov–Smirnov test >0.15; mean, 1.00; standard deviation, 0.22; 95% confidence interval, 0.84–1.38). The odds ratio of the interaction term for the three affected prefectures (Iwate, Miyagi, and Fukushima Prefectures) × cohort in pooled data (odds ratio 1.56) was in the 99th percentile of the distribution and corresponded to *P* = 0.014 in a two-sided test of the permutation test.

**Table 2:** Adjusted odds ratios and 95% confidence intervals for the onset of overweight at follow-up in young children without overweight at baseline.

	Cohort 1 (Ne/Np = 875/40,046)		Cohort 2 (Ne/Np = 1235/53,492)		Pooled (Ne/Np = 2110/93,538)	
	OR (95% CI)	*P*	OR (95% CI)	*P*	OR (95% CI)	*P*
School location × cohort	–		–		1.56 (1.09–2.23)	0.015
Cohort (Cohort 2 = 1, Cohort 1 = 0)	–		–		1.08 (0.98–1.20)	0.111
School location (three prefectures = 1, other prefectures = 0)	0.80 (0.60–1.06)	0.12	1.25 (1.01–1.55)	0.040	0.80 (0.60–1.07)	0.130
Sex (boy = 1, girl = 0)	1.34 (1.16–1.55)	0.0001	1.35 (1.19–1.53)	<0.0001	1.34 (1.22–1.48)	<0.0001
Age (per 1 year increase)	2.64 (2.04–3.42)	<0.0001	2.30 (1.84–2.86)	<0.0001	2.43 (2.06–2.88)	<0.0001
History of disease (present = 1, absent = 0)	0.98 (0.77–1.25)	0.87	0.93 (0.76–1.13)	0.46	0.95 (0.81–1.10)	0.492
Obesity index at baseline (per 1% increase)	1.46 (1.43–1.49)	<0.0001	1.45 (1.43–1.48)	<0.0001	1.46 (1.44–1.47)	<0.0001

CI, confidence interval; Ne/Np, number of event/number of population; OR, odds ratio.

All variables in the table were simultaneously included in the logistic regression model as dependent variables. School location × cohort was an interaction term of school location (the three prefectures = 1, the other prefectures = 0) and cohort (Cohort 2 = 1, Cohort 1 = 0).

We repeated the following analyses as sensitivity analyses: setting the exposure group as children with their school location in (1) Iwate Prefecture, in (2) Miyagi Prefecture, or in (3) Fukushima Prefecture instead of the three prefectures combined, (4) overweight defined as an obesity index of >10%, (5) follow-up measurement set to 1.5 years after baseline, and (6) follow-up measurement set to 2.0 years after baseline. All sensitivity analyses were confirmatory, though in analyses of (1), (2), and (3), the number of events in exposure group was <25 and odds ratios in cohort 2 and pooled data were not statistically significant (Table 3).

**Table 3:** Sensitivity analyses for the onset of overweight in young children without overweight at baseline.

	Cohort 1				Cohort 2				Pooled	
	Number of events^b^	Number of population^b^	OR^a^ of school location (95% CI)	*P*	Number of events^b^	Number of population^b^	OR^a^ of school location (95% CI)	*P*	OR^a^ of school location × cohort (95% CI)	*P*
(1) Iwate Prefecture	13/875	591/40,046	0.70 (0.39–1.26)	0.23	27/1235	863/53,492	1.35 (0.88–2.06)	0.17	1.93 (0.93–4.00)	0.23
(2) Miyagi Prefecture	24/875	1234/40,046	0.68 (0.44–1.05)	0.09	47/1235	1624/53,492	1.07 (0.78–1.48)	0.67	1.56 (0.91–2.69)	0.11
(3) Fukushima Prefecture	23/875	730/40,046	1.14 (0.71–1.81)	0.60	41/1235	1064/53,492	1.41 (0.99–2.00)	0.06	1.23 (0.69–2.22)	0.48
(4) Overweight was defined as an obesity index of >10%.	93/1343	2334/37,490	0.92 (0.73–1.16)	0.47	177/1921	3282/50,240	1.32 (1.11–1.57)	0.002	1.42 (1.07–1.89)	0.017
(5) Follow-up was 1.5 years after baseline.	89/1255	2561/39,819	0.85 (0.67–1.08)	0.17	176/1806	3544/53,156	1.35 (1.13–1.62)	0.0009	1.60 (1.19–2.15)	0.002
(6) Follow-up was 2.0 years after baseline.	132/1799	2530/39,812	0.93 (0.77–1.14)	0.50	234/2676	3523/52,589	1.18 (1.02–1.38)	0.032	1.26 (0.98–1.62)	0.067

CI, confidence interval; OR, odds ratio.

a Adjustments applied for sex, age, history of disease, and obesity index at baseline. School location was defined as (1) Iwate Prefecture = 1, and the other prefectures = 0, (2) Miyagi Prefecture = 1, and the other prefectures = 0, (3) Fukushima Prefecture = 1, and the other prefectures = 0, and (4–6) the three prefectures = 1, the other prefectures = 0. School location × cohort was an interaction term of school location and cohort (Cohort 2 = 1, Cohort 1 = 0).

b Number of events, and number of subjects were the number of events and population in the three prefectures/all prefectures in Japan, respectively.

Among the 49,174 children in cohort 2 without missing values for questionnaire items on severe disaster experience, we compared the onset of overweight at follow-up among children with (n = 503) and without (n = 48,671) severe disaster experience. The onset of overweight tended to be more common in children with severe disaster experience than those without (n = 16/503, 3.18% vs. n = 1115/48,671, 2.29%, *P* = 0.18). When overweight was defined as an obesity index of >10%, the onset of overweight was significantly more likely in children with severe disaster experience than in those without (n = 28/460, 6.09% vs. n = 1725/45,731, 3.77%, *P* = 0.014). For specific severe disaster experience, “House destroyed” (n = 15/196, 7.65% vs. n = 1738/45,995, 3.78%, *P* = 0.013), “Tsunami” (n = 11/100, 11.00% vs. n = 1742/46,091, 3.78%, *P* = 0.002), and “Moving house” (n = 5/49, 10.20% vs. n = 1748/46,142, 3.79%, P = 0.002) were significant risk factors for overweight when overweight was defined as an obesity index of >10%.

For the onset of underweight, as defined by an obesity index of <−15%, we did not find any significant odds ratios in the three affected prefectures in either cohort 1 or cohort 2. Similarly, the odds ratios of children with severe disaster experience were not significant.

## Discussion

We investigated the onset of overweight and underweight among young children aged 3.5–4.5 years after the Great East Japan Earthquake without overweight and underweight, respectively, at baseline all over Japan. We found that the odds ratio for the onset of overweight at a follow-up of 1 year after baseline (about 0.5 years after the earthquake) in the three prefectures of Iwate, Miyagi and Fukushima, which were the areas most severely damaged by the earthquake, was significantly higher in cohort 2 (children who were in nursery school at the time of the earthquake), but not in historical control of 53,492 children (cohort 1), compared to children in other prefectures. These findings were formally confirmed in analysis of pooled data from the two cohorts; the odds ratio of the interaction term of school location × cohort was significant, but that of the three prefectures was no longer significant, suggesting that the earthquake was associated with the onset of overweight in young children in the three affected prefectures. To our knowledge, this is the first study to reveal that a huge earthquake could be associated with overweight in young children. In relation to underweight, we did not find any significant results.

In our present study, the odds ratio for the onset of overweight in the three prefectures most affected by the earthquake was 1.25. Considering the large size of the population at risk, this mild effect size of the impact of earthquake on overweight should be recognized as a serious problem. Obviously, a huge earthquake can occur in a wide range of areas and affect a large number of victims indiscriminately. Therefore, even a moderate increase in disease risk could be a major health problem from a population viewpoint. A systematic review showed that overweight children become overweight adults, suggesting that childhood overweight or obesity persists into adulthood.^13^ Childhood rapid weight gain^14^ and continuity of overweight^15^ were also associated with development of cardiovascular disease and diabetes mellitus in adulthood. Therefore, the health status of young children in severely damaged areas should be carefully monitored, and anthropometric measurements should be taken continuously during not only childhood, but also into adulthood.

The association between physical activity and obesity in young children has been investigated.^16^ Time spent in outdoor play was associated with physical activity levels, as assessed using accelerometers, in young children.^17^, ^18^ However, after the Great East Japan Earthquake, reduction in time spent in outdoor play of children had been a concern in Iwate, Miyagi, and Fukushima Prefectures. Play space was decreased after the disaster: in municipalities located along the Pacific coast, schoolyards and parks became temporary housing sites. These changes in environmental factors could negatively influence children's physical activity.^19^ Some parents prohibited their children to go to outdoor play spaces because of increased construction vehicle traffic on community roads associated with reconstruction. Especially in Fukushima Prefecture, children's outdoor activities were also restricted due to region-wide radiation contamination after the nuclear accident following the earthquake. These environmental factors of reduction in children's play space seemed to be impermanent. This might partly explain why the odds ratio for the onset of overweight in the three affected prefectures tended to decrease with the length of follow-up period, with odds ratios of 1.25, 1.35, and 1.18 for 1-year, 1.5-year, and 2-year follow-up, respectively (Table 2, Table 3). Because we only have data until October 2012 in our study, we cannot extend the follow-up period of our study. Further studies or surveys are needed in order to elucidate the important question raised in our study, including whether or not the observed high incidence of overweight after the earthquake in young children in the three affected prefecture persists over the years to come.

Underweight in children after disasters in developing countries has been a major concern.^6^, ^7^ On the contrary, there is little data on disaster-related underweight among children in developed countries. In the present study, we did not detect any increase in underweight among children after the earthquake; the odds ratio of the onset of underweight at follow-up in the three affected prefectures was not significant among young children without underweight at baseline. The null association between underweight and experience of earthquake among children in developed countries may be due to the highly developed social environment of developed countries, including stockpiling of emergency food, developed transportation networks, national attention due to intense press coverage, and many offers of assistance from volunteer and highly developed public health organizations. Indeed, after the Great East Japan Earthquake, there was no massive death from starvation or epidemics of severe infection, such as cholera, typhoid fever, and dysentery.^20^

This study has several limitations. First, the participation rate was significantly different between three affected prefectures (30.3%) and other prefectures (14.6%, chi-square *P* < 0.0001). Schools with more health-conscious administrators or more manpower might have been more likely to participate in our survey. Nevertheless, in cohort 1, the onset of overweight was similar between the three affected prefectures and the other prefectures (2.35% vs. 2.17%; *P* = 0.53). Therefore, we believe that the effects of the low participation rate in other prefectures did not affect our results. Second, our project was a retrospective cohort study. However, our results are not thought to be affected by recall bias because we collected pre-existing data from scheduled anthropometric measurements accumulated in each nursery school. Furthermore, nursery teachers were licensed by the government and graduated from schools certified by the Ministry of Health, Labour and Welfare. Therefore, nursery teachers have adequate training and skills to collect accurate child health information, and we believe that the anthropometric measurements used in the present study had sufficient accuracy for the analysis. Third, in line with school health statistics^10^ from the Ministry of Education, Culture, Sports, Science and Technology, we observed that both BMI and obesity index at the baseline were higher in the three affected prefectures than in the other prefectures. Therefore, to minimize the effect of the high baseline obesity index, we adjusted the obesity index at baseline in logistic analyses and collected children's data of historical cohort. However, we cannot deny the possibility of unknown confounding factors that lead to overweight in the three affected prefectures as regional characteristics. Our results derived from the three affected prefectures should be carefully applied to other regions where prevalence of obesity or overweight in children are not high.

Presently, young children in areas severely damaged by the Great East Japan Earthquake have an increased risk of childhood overweight compared to children in other prefectures of Japan, and this risk could persist until adulthood; therefore, the health status and anthropometric measurements of these children should be carefully and continuously monitored.

## Conclusion

Onset of overweight in young children was significantly higher in the three prefectures most affected by the Great East Japan Earthquake than in other prefectures after the disaster.

## Conflicts of interest

None declared.
